# The TrmB family: a versatile group of transcriptional regulators in Archaea

**DOI:** 10.1007/s00792-014-0677-2

**Published:** 2014-08-13

**Authors:** Antonia Gindner, Winfried Hausner, Michael Thomm

**Affiliations:** Department of Microbiology and Archaea Center, University of Regensburg, Universitätsstraße 31, 93053 Regensburg, Germany

**Keywords:** Archaea, Transcriptional regulators, Sugar metabolism, TrmB family, Transcription

## Abstract

Microbes are organisms which are well adapted to their habitat. Their survival depends on the regulation of gene expression levels in response to environmental signals. The most important step in regulation of gene expression takes place at the transcriptional level. This regulation is intriguing in Archaea because the eu-karyotic-like transcription apparatus is modulated by bacterial-like transcription regulators. The transcriptional regulator of mal operon (TrmB) family is well known as a very large group of regulators in Archaea with more than 250 members to date. One special feature of these regulators is that some of them can act as repressor, some as activator and others as both repressor and activator. This review gives a short updated overview of the TrmB family and their regulatory patterns in different Archaea as a lot of new data have been published on this topic since the last review from 2008.

## Introduction

On the basis of 16S RNA analysis the third domain of life representing the Archaea was proposed by Woese, Kandler and Wheelis in 1990 (Woese et al. 1990). This was confirmed later by studies of the Archaeal biochemistry and molecular biology. What distinguishes Archaea from the other two domains is the fact that they possess both bacterial and eukaryal properties. On the one hand, they have transcription, translation and DNA replication machineries which are similar to those of eukaryotic organisms (Keeling and Doolittle 1995; Langer et al. 1995; Dennis 1997; Grabowski and Kelman 2003). On the other hand, many genes involved in metabolic processes are more similar to those encoded in bacterial genomes (Koonin et al. 1997).

Already in the late 1970s it was discovered that Archaea have a multi-subunit RNA polymerase (RNAP) homologous to the eukaryotic RNAP II (Zillig et al. 1979; Huet et al. 1983). In addition, the initiation factors TATA box binding protein (TBP) and transcription factor B (TFB) (Hausner et al. 1996; Ouhammouch et al. 2003), both related to their eukaryotic representatives, are important components of the Archaeal transcription machinery. An Archaeal promoter consists of a transcription factor B recognition element (BRE) (Littlefield et al. 1999) comprising two adenines at −34/33 and a TATA box (Thomm and Wich 1988) about −26/27 base pairs upstream of the transcription start site. Furthermore, an initiator motif (INR) mostly consisting of a pyrimidine-purine di-nucleo-tide at the transcription start site is essential for initiation (Reiter et al. 1990; Hausner et al. 1991). Most DNA-binding proteins with known gene-regulatory function resemble bacterial activators or repressors (Kyrpides and Ouzounis 1999). Just a small minority of these transcriptional regulators is homologous to eukaryotic proteins (Aravind and Koonin 1999; Bell and Jackson 2001). However, interactions between bacterial-like regulators and the eukaryotic-like basal transcription machinery are still a matter of debate (Di Fiore et al. 2009).

Most of the DNA-binding transcription factors (TFs) are repressors but also activators or proteins with both activities are known (Bell 2005; Geiduschek and Ouhammouch 2005; Grohmann and Werner 2011; Peeters et al. 2013). A family which contains all of the three possible TF combinations—repressors, activators or both—is the TrmB family. Recently published data about the distribution of proteins that possess a TrmB-like DNA–binding domain (DBD) (Pfam–ID: PF01978) and/or a TrmB-like effector-binding domain (EBD) (Pfam–ID: PF11495) show a complete spreading all over the three domains of life (Maruyama et al. 2011). The DBD belongs to the helix-turn-helix (HTH) motifs which is prevalent in many Bacteria and Archaea (Pérez-Rueda and Janga 2010). However, TrmB proteins that possess both a DBD and an EBD are more common in Archaea. Most of them were found in the phylum Euryarchaeota but some exist also in Crenarchaeota as well as one in Thaum-, Nano- and Kor-archaeota, respectively (Maruyama et al. 2011).

### Members of the TrmB family in Euryarchaeota

Proteins of the TrmB family can be found in 13 genera in the Euryarchaeota. The orders Thermococcales, Halobacteriales and Thermoplasmatales encode altogether 41 representatives in their genomes (Maruyama et al. 2011). In thermophilic Archaea TFs of the TrmB family are involved in the regulation of sugar metabolism, especially for maltose and glucose processing (van de Werken et al. 2006; Kanai et al. 2007; Lee et al. 2008). The gene loci which encode TrmB also flank genes that code for the maltose and/or trehalose transporters. In contrast, these genetic loci are not adjacent to VNG1451C, the halobacterial version of TrmB in *Halobacterium salinarum* (Schmid et al. 2009) or MreA, the regulator of methanogenic pathways in *Methanosarcina acetivorans* (Reichlen et al. 2012). In recent years, more and more TrmB-like proteins were detected in different Archaea and so far they are likely to play an important role in diverse metabolic processes. The results indicate that TrmB arose in one of these organisms and has subsequently been disseminated afterwards by horizontal gene transfer to other Archaea or Bacteria (DiRuggiero et al. 2000). This review focusses on the best studied representatives of TrmB proteins in the Euryarchaeota *Pyrococcus furiosus, Thermococcus litoralis*, *Thermococcus kodakarensis*, *Halobacterium* *salinarum* and *Methanosarcina* *acetivorans.* Some of the characteristics of the TrmB family are summarized in Table 1.

**Table 1:** Overview of the discussed TrmB homologs

Euryarchaeota								Cren-archaeota		
Thermococcales								Halobacteriales	Methano-sarcinales	Sulfolobales
	*Pyrococcus furiosus*				*Thermococcus litoralis*	*Thermococcus kodakarensis*		*Halobacterium salinarum*	*Methanosarcina acetivorans*	*Sulfolobus acidocaldarius*
	TrmB	TrmBL1	TrmBL2	TrmBL3	TrmB	Tk1769/Tgr/TrmBL1	Tk0471/TrmBL2	VNG1451C	MreA	MalR
aa	338	341	264	264	338	341	264	360	139	349
kDa	38.8	39.4	30.6	30.2	38.8	39.4	30.8	39.4	15.9	40.3
structure	Dimer	Tetramer/Octamer			Dimer					
N-Terminus	DBD	DBD	DBD	DBD	DBD	DBD	DBD		DBD	DBD
	wHTH	HTH	HTH		wHTH	HTH	HTH	wHTH	HTH	HTH
DNA-binding aa	Y50, D51	Y49, D50	Y50, D51	Y50, E51	Y50, D51	Y49, D50	Y50, D51	Y61, D62	S66, L67	Y51, N52
C-Terminus	EBD	EBD	–	–	EBD	EBD	–	EBD	–	EBD
Sugar binding aa	G320, E326	G320, E326	–	–	G320, E326	G324, E330	–	G337, E343	–	G320, E326
Acting as	Repressor	Repressor, activator	Putative chromosomal protein		Repressor	Repressor, activator	Repressor, chromosomal protein	Repressor, activator	Repressor, activator	Activator
Inducers	Maltose sucrose maltotriose maltodextrins trehalose	Maltose maltotriose fructose			Maltose trehalose	Maltotriose		Glucose glycerol		
Co-repressors	Glucose (TM,MD) maltotriose (TM) maltose (MD)									
Binding motif	TACTN_3_AGTA (TM) TACT (MD)	TGM: TATCACN_5_ GTGATA			TACTN_3_AGTA (TM)	TGM: TATCACN_5_ GTGATA	Coding and intergenic regions	TACTN_7–8_ GAGTA		Two repeats ATAATACT
						Auto-regulation		Auto-regulation		Auto-regulation proposed

*aa* amino acids, *kDa* kilodalton, *DBD* DNA-binding domain, *EBD* effector binding domain, *(w)HTH* (winged) helix-turn-helix motif, *TM* trehalose/maltose operon, *MD* maltodextrin operon (modified after Lee et al. 2003, 2005, 2007a, 2007b, 2008; Krug et al. 2013; van de Werken et al. 2006; Kanai et al. 2007; Maruyama et al. 2011; Schmid et al. 2009; Reichlen et al. 2012; Wagner et al. 2013)

### TrmBs in the Thermococcales

A sequence alignment of *Pyrococcus furiosus* TrmB to TrmB-like proteins in the order Thermococcales revealed five different clusters of TrmB-like proteins (Lee et al. 2007b). An overview of the distribution of TrmB family proteins is given in Table 2. However, none of the TrmB variants is present in all organisms. This alignment showed that *P.* *furiosus* contains four different TrmB-like proteins (Table 2). A closer look at their amino acid sequence showed a 29 % sequence identity between TrmB and TrmBL1 (PF0124). Especially the N-terminus, which contains a HTH motif as DBD in TrmB, is highly conserved (45 % sequence identity). In addition, a C-terminal EBD can be found in TrmBL1. TrmBL2 (PF0496) also possesses a high sequence identity to the N-terminus of TrmB indicating DNA binding but the C-terminal sequence is lacking 70 amino acids containing the sugar binding domain. The same is true for TrmBL3 (PF0661) (Lee et al. 2007b).

**Table 2:** Distribution of TrmB family proteins in the Thermococcales

	TrmB	TrmBL1	TrmBL2	TrmBL3	TrmBL4
*P. furiosus*	PH1743	PF0124	PF0496	PF0661	−
*T. litoralis*	Q7LYW4 (100 %)	−	−	−	−
*T. kodakarensis*	−	TK1769 (67 %)	TK0471 (82 %)	−	−
*P. horikoshii*	PH1034 (71 %)	−	PH0799 (92 %)	−	PH0751
*P. abyssi*	−	−	PAB0838 (91 %)	−	−

The values in percent show the identity of the amino acid sequence in comparison to the corresponding protein of *Pyrococcus furiosus*. A minus sign states that the appropriate protein is missing in the organism. The identity among the different paralogous members was between 22 and 30 % (modified after Lee et al. 2007b)

**Fig. 1:**
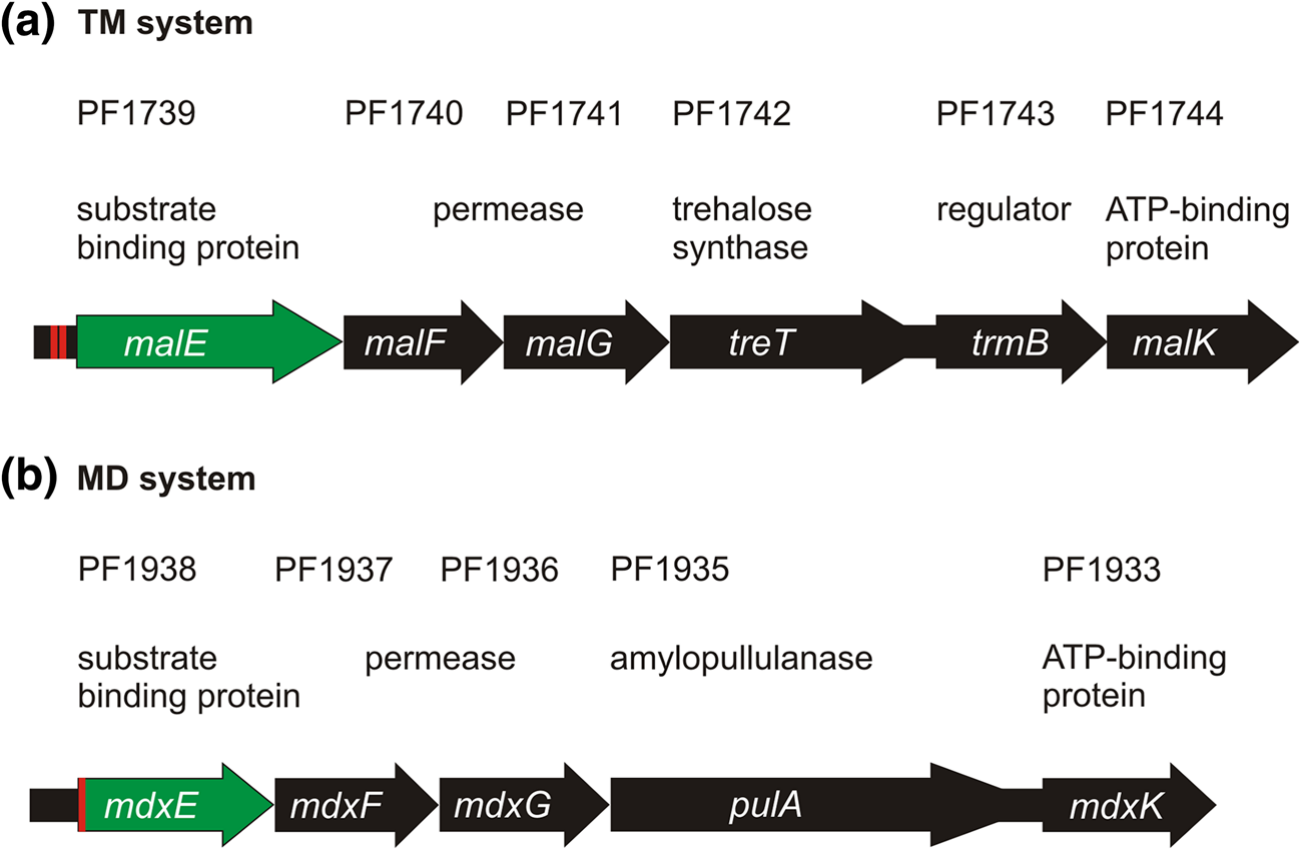
The gene clusters of the trehalose/maltose (TM) and the maltodextrin (MD) operon. **a** The TM gene cluster encoding the binding protein-dependent ABC transporter for trehalose/maltose, a trehalose synthase and TrmB is shown. The *red lines* mark the palindromic binding sequence of TrmB (TACTN_3_AGTA) at the promoter region of *malE* (*green*). **(b)** The MD gene cluster encoding the binding protein-dependent ABC transporter for maltodextrin and an amylopullulanase is shown. A separate regulator is missing in the MD operon. The *red line* tags the first half of the binding sequence palindrome (TACT) (modified after Lee et al. 2008)

The presence of TrmB in *Pyrococcus furiosus* seems to be a result of lateral gene transfer between *Thermococcus* *litoralis* and *P.* *furiosus* (Imamura et al. 2004). The gene cluster containing TrmB and the ATP binding cassette (ABC) transport system for maltose and trehalose (TM operon) is located in a 16 kb region in *T.* *litoralis* which is flanked by one remaining IS element (DiRuggiero et al. 2000). The cluster is composed of five genes, *malE malF malG treT trmB* and *malK* forming a binding protein-dependent ABC transporter for maltose and trehalose with nearly identical sequence to the *P.* *furiosus* TM operon (Fig. 1a) (Xavier et al. 1996; Horlacher et al. 1998; Greller et al. 1999; Xavier et al. 1999; DiRuggiero et al. 2000; Lee et al. 2003; Qu et al. 2004).

**Fig. 2:**
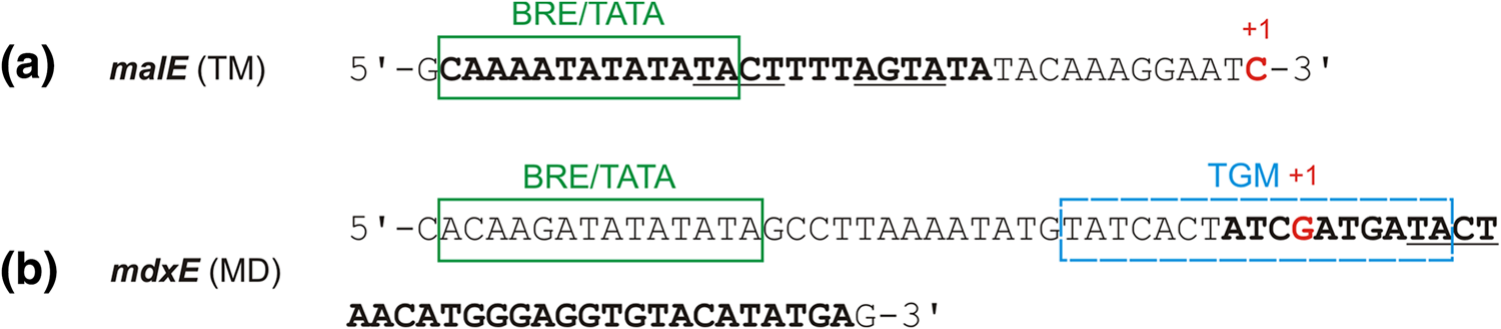
Binding sites of *Pfu* TrmB at the first genes of the TM (*malE*, **a**) and MD (*mdxE*, **b**) operon. The binding sites are shown in *bold letters*. +1 represents the transcription start site. The promoter region of the *malE* gene includes a perfect palindrome (*underlined*), whereas the promoter region of *mdxE* just contains the first half of the palindrome. The B recognition element (BRE) and the TATA-*box* are shown in *green boxes*. The Thermococcales glycolytic motif (TGM, van de Werken et al. 2006), just present in the *mdxE* promoter, is in a *blue box* (modified after Lee et al. 2008)

**Fig. 3:**
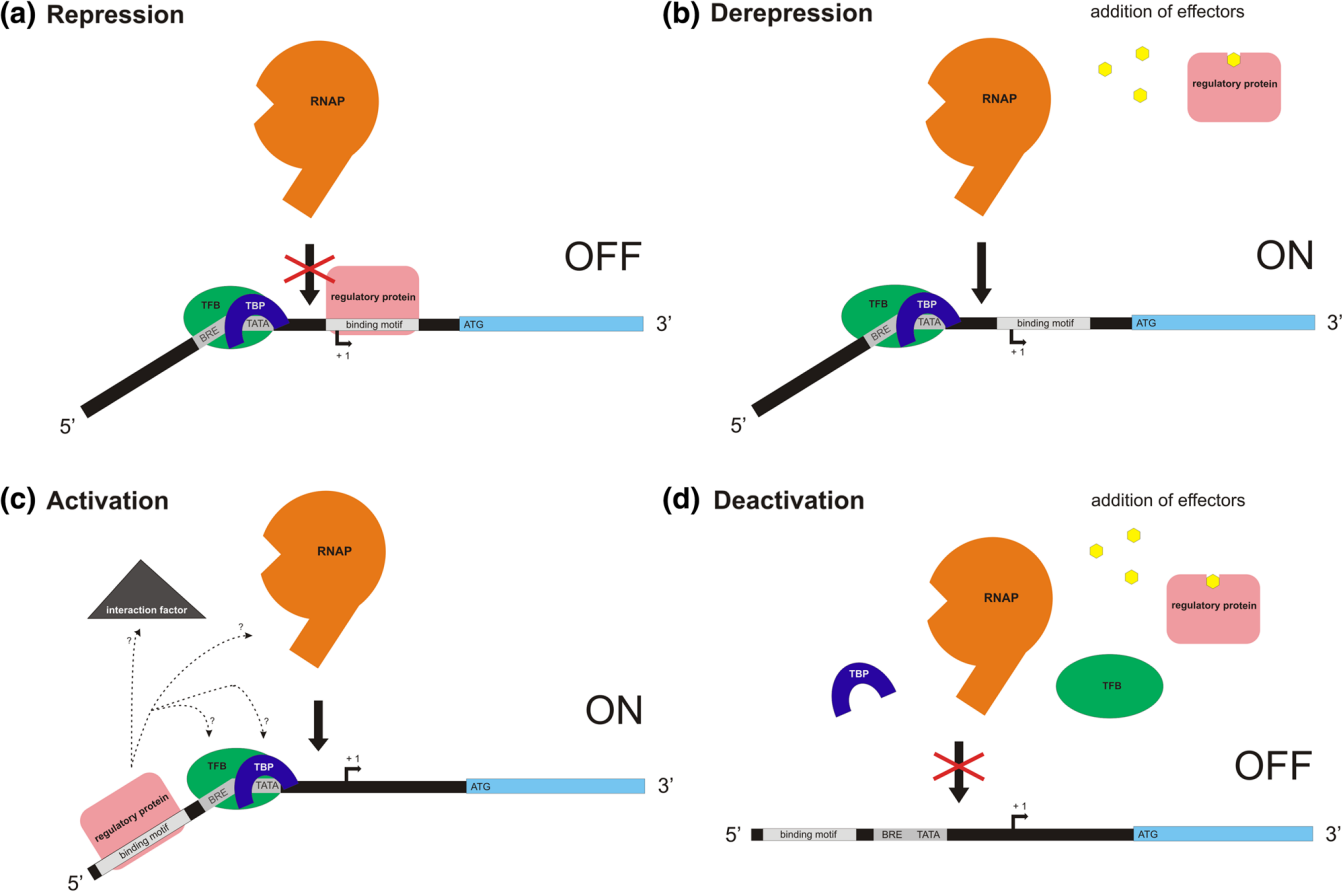
Models of the regulation mechanism of *P.* *furiosus* TrmB (**a** and **b**) and TrmBL1 (**a**–**d**), *T.* *littoralis* TrmB (**a** and **b**), *T.* *kodakarensis* Tgr (**a**–**d**) and *H.* *salinarum* VNG1451C (**a**–**d**) for genes where the proteins are acting as repressor and/or as activator. All binding motifs are palindromic inverted repeats with *cis*-regulatory sequences in front of the BRE/TATA box or at the transcription start site (+1). The binding motif for *Pfu* TrmB and *Tli* TrmB at the TM promoter is TACT-N_3_-AGTA, at the MD operon of *P. furiosus* it is just the TACT sequence. *Pfu* TrmBL1 and *Tk* Tgr bind to TATCAC-N_5_-GTGATA (TGM), the binding motif in *H.* *salinarum* is TACT-N_7-8_-GAGTA. TBP, TATA binding protein; TFB, transcription factor II B; RNAP, RNA polymerase (modified after Lee et al. 2008 and Kanai et al. 2007)

*Pfu* TrmB acts as transcriptional repressor for genes of the TM operon. A palindromic sequence—TACTNNNAGTA—was detected as TrmB recognition site at the BRE/TATA box of the *malE* gene (Fig. 2a) (Lee et al. 2003). Repression takes place by preventing the recruitment of the RNAP to the *malE* promoter (Fig. 3a). Maltose and trehalose act as inducers by detaching TrmB from the recognition site (Fig. 3b) (Lee et al. 2003). These inducers, however, show a difference in their binding affinity. Maltose is bound to purified TrmB in a positive cooperative fashion whereas binding of trehalose shows no sigmoidal binding behavior (binding of trehalose is at least 20-fold lower than of maltose) (Lee et al. 2008). This could be explained in terms of their transport and metabolism. Studies in *T.* *litoralis* have shown that both maltose and trehalose share an equal rate and a high affinity in transportation but their metabolism is quite different. Whereas maltose is metabolized quickly, trehalose is just slowly metabolized and accumulates to high internal concentrations when it is present in the medium (Lamosa et al. 1998).

Nonetheless, *Pfu* TrmB is also a repressor for genes of a separate maltodextrin ABC transporter (MD operon) (Fig. 1b). The MD operon is composed of the genes *mdxE mdxF mdxG pulA* and *mdxK* (Koning et al. 2002; Lee et al. 2003). This MD operon was identified with the same gene arrangement in *T.* *litoralis* as well. According to individual genes the sequence identity varies from 51 to 83 % between *T.* *litoralis* and *P.* *furiosus* (Imamura et al. 2004). DNA sequence comparison revealed that *malK* was the result of a duplication of the *mdxK* gene before the *mal* gene cluster was transferred from *T.* *litoralis* to *P.* *furiosus* (Imamura et al. 2004).

In contrast to the *Pfu* *malE* promoter of the TM operon, the *Pfu* *mdxE* promoter contains only the first half of the TrmB recognition sequence (TACT) at the transcription start site (Fig. 2b). The second half of the inverted repeat is missing and in vitro transcription experiments showed a reduced binding affinity of TrmB to the MD operon (Lee et al. 2005). The *Pfu malE* gene is completely repressed in the presence of 0.2 µM TrmB whereas *Pfu mdxE* is not repressed until an amount of 1.6 µM. Furthermore, binding of *Pfu* TrmB is controlled by differential sugar binding activity. Maltose, sucrose, maltotriose, maltodextrins and trehalose are bound with decreasing order of affinity. At the TM promoter TrmB can be released using maltose and trehalose as inducers. In contrast, bound TrmB at the MD promoter can be released only in the presence of maltotriose, maltodextrins and sucrose, but not with maltose or trehalose (Lee et al. 2005). Later experiments showed that glucose acts as a co-repressor at the TM and MD operon, causing stronger repression when maltose and maltotriose is present, respectively (Lee et al. 2007a). Indeed, *P.* *furiosus* is not able to transport glucose but can metabolize it. Glucose originates from the cytoplasmic dextrin metabolism and its presence in excess represses both the TM and the MD system and stops the uptake of glucose-producing sugars like maltose, trehalose or maltodextrins even in the presence of inducers (Lee et al. 2008). In addition, the presence of both, maltotriose and maltose, at the MD operon at the same time led to stronger repression showing also a co-repressor activity of maltose for the MD system (Lee et al. 2007a).

TrmB of *P.* *furiosus* (PF1743) as well as of *T.* *litoralis* (Q7LYW4) consists of 338 amino acids, forms a protein of 38.8 kDa and occurs at room temperature as a dimer (Table 1) (Lee et al. 2003). Hitherto, the *Pyrococcus* TrmB is the only one with a described crystal structure (Krug et al. 2006, 2013). As the conserved sequences of the other TrmB representatives argue for a similar structure we review the structural details of *Pyrococcus* TrmB in more detail hereafter.

**Fig. 4:**
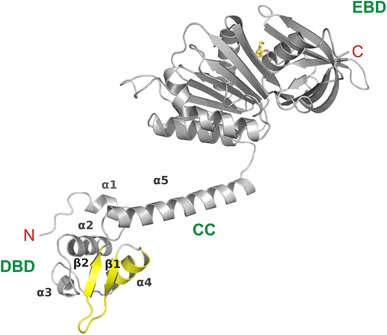
The structure of *Pyrococcus furiosus* TrmB with bound sucrose in *yellow* wireframe (ribbon presentation). The N-terminal DNA-binding domain (DBD) consists of a winged helix-turn-helix motif. Helix α4 represents the DNA recognition helix. The wing and the recognition helix are colored *yellow*. Helices (α) and strands (β) are consecutively numbered. Helix α5 (CC) and a short linker connect the DBD to the sugar/effector binding domain (EBD) harboring sucrose (in *yellow*) (taken from Krug et al. 2013 with permission from the authors and the publisher)

**Fig. 5:**
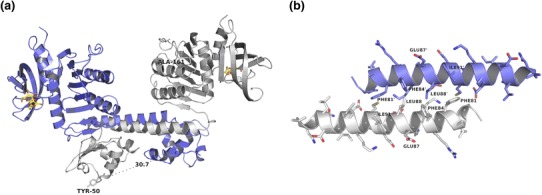
**a** Structure of the *Pyrococcus furiosus* TrmB dimer in ribbon presentation and bound sucrose in *yellow* wireframe. The structure represents the dimer created by -X, Y-X, 2/3-Z crystallographic symmetry operation. One monomer is colored *grey*, the other *mauve*. The protein presumably builds a dimer by forming a coiled coil of the CC helices of the two monomers. The dimer can be considered as a result of domain swapping of the DBDs between two copies of an ancestral protein consisting of the EBD and the DBD with the CC helices as a hinge loop. The distances between the two recognition helices (α4) are indicated. **b** Zooming of the coiled-coil formed by two crystallographic counterparts of CC in ribbon presentation with side chains in stick representation. The hydrophobic residues Phe_81_/Ile_91′_, Phe_84_/Leu_88′_, Leu_88_/Phe_84′_ and Ile_91_/Phe_81′_ are represented in a zipper-like arrangement (taken from Krug et al. 2013 with permission from the authors and the publisher)

The structure is split into an N-terminal DBD and a C-terminal EBD (Fig. 4). The N-terminal DBD contains a winged-helix-turn-helix (wHTH) motif composed of four α-helices Dα1–α4 and two β-strands Dβ1–β2. Via the Dα4-helix which is responsible for DNA recognition and its counterpart Dα4′ in the dimeric protein, TrmB is able to bind to two contiguous major grooves of duplex palindromic B-DNA (Krug et al. 2013). Especially tyrosine at position 50 and to a lesser extent aspartic acid at position 51 thereby seem to play an important role concerning DNA binding (Fig. 5a) (Lee et al. 2007a). The wHTH motif is followed by an amphipathic helix α5 containing a coiled-coil (CC) motif which advances dimerization. Pairs of hydrophobic amino acids in this CC and a counterpart CC’, Phe81/Ile91′ and Phe84/Leu88′ as well as their correspondent part, provide a basis for interaction between two monomer proteins to form a dimer (Fig. 5b) (Krug et al. 2013).

**Fig. 6:**
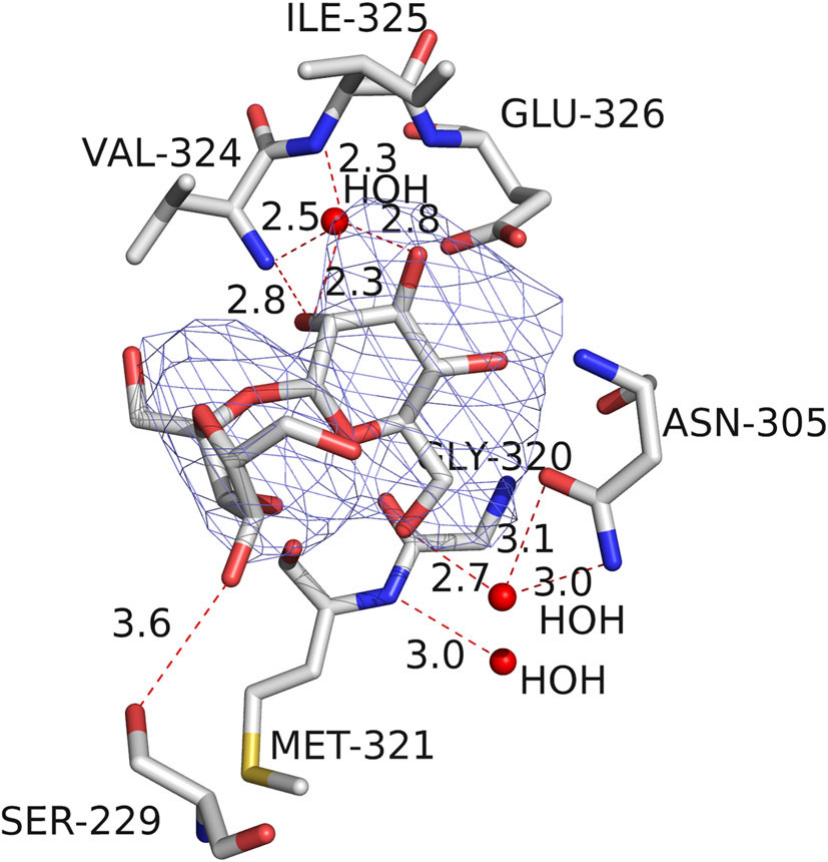
The sugar binding domain (EBD) of *Pyrococcus furiosus* TrmB in complex with sucrose. The TrmB residues which interact with sucrose as well as the distances of potential hydrogen bonds in Å units are indicated. The omit electron density map of sucrose is shown at the 5 σ level (taken from Krug et al. 2013 with permission from the authors and the publisher)

The C-terminus represents an EBD consisting of two subdomains. The first subdomain is built up of an eight-stranded sheet Eβ1-8 flanked by two large helices Eα1–2 on the one side and one large helix Eα3 crossing the β-sheet at the other side. The second subdomain, which is connected to the first one by a short hinge, forms a strand Eβ9, a helix Eα4 and an irregular flattened seven-stranded β-barrel with its axis parallel to the strands of the first subdomain (Krug et al. 2006). The sugar recognition helix Eα3 is on the surface of the cleft between these two subdomains. Regardless of whether maltose or sucrose is bound, their nonreducing glucosyl moieties interact both with the same six amino acids of the second C-terminal subdomain—Asn_305_, Gly_320_, Met_321_, Val_324_, Ile_325_ and Glu_326_ (Fig. 6). Just the orientation of maltose or sucrose in the bound state is different. The reducing glucosyl moiety of maltose contacts a seventh amino acid (Ser_229_), which can be found in the sugar recognition helix Eα3, via hydrogen bonding. However, it is not clear whether or not the fructosyl moiety of sucrose acts analogue (Krug et al. 2013).

In 2006 van de Werken et al. described a conserved sequence motif named TGM (Thermococcales glycolytic motif) at promoter sites of genes encoding glycolytic enzymes as well as several other genes involved in sugar metabolism of *P.* *furiosus* and *T.* *kodakarensis*. The TGM consists of a conserved inverted repeat interspaced by five nucleotides (TATCAC-N_5_-GTGATA). This putative *cis*-acting element indicated a common transcriptional control of the respective genes. The inverted repeat, however, is missing in *Pyrococcus abyssi* and *Pyrococcus horikoshii* thereby indicating that their reduced catabolic effectiveness does not require such a regulatory system (van de Werken et al. 2006).

Since the genome of *T.* *kodakarensis* contains neither a TM operon nor an ortholog of TrmB, it was hypothesized that another global regulator capable of recognizing the TGM exists. Moreover, an ortholog of the MD operon (TK1771-TK1775) as well as two paralogs to TrmB-like genes (TK0471 and TK1769) can be found (Table 1) with TK1769 being adjacent to the MD operon (Kanai et al. 2007). Sequence analysis of TrmB-like regulators which may be involved in recognizing the TGM identified PF0124 from *P.* *furiosus* (TrmBL1, TrmB-like 1) and TK1769 from *T.* *kodakarensis* (Tgr, Thermococcales glycolytic regulator), respectively, as the expected global regulators with a 67 % amino acid identity (Table 2) (Lee et al. 2007b).

TrmBL1 and Tgr are TGM-recognizing global sugar-sensing transcription regulators of genes coding for glycolytic and gluconeogenic enzymes as well as for sugar transport systems. TrmBL1 responds to maltose, maltotriose and fructose which act as inducers to release DNA-bound protein. Tgr just responds to maltotriose as inducer. Furthermore, both proteins play a binary regulatory role: they are both activators for certain gluconeogenic genes and repressors for glycolytic enzymes (Kanai et al. 2007; Lee et al. 2008). If both proteins act as repressors at TGM containing promoters, the TGM is situated downstream of the BRE/TATA box, mostly overlapping the transcription start site and interfering with the recruitment of RNAP (Fig. 3a) (Kanai et al. 2007; Lee et al. 2007b) like it was already shown for MDR1 of *Archaeoglobus fulgidus* (Bell et al. 1999) or Phr of *P.* *furiosus* (Vierke et al. 2003).

This mechanism is also proposed for a potential glycolytic regulon of *P.* *furiosus* including a phospho-sugar mutase (PF0588) and three α-amylase encoding genes (PF0272, PF0478 and PF0477), as all of the corresponding promoters contain a TGM downstream of the BRE/TATA box (van de Werken et al. 2006). In contrast, if TrmBL1 and Tgr act as activators, the TGM is found upstream of the BRE/TATA box. Such an activation mechanism was already shown for Ptr2 from *Methanococcus jannaschii* (Ouhammouch et al. 2003) which interacts with TBP or TFB-RF1 (PF1088) from *P.* *furiosus* (Ochs et al. 2012) which interacts with TFB. A similar activation mode via interaction with TBP, TFB, RNAP or even a yet unknown factor is also proposed for TrmBL1 (Fig. 3b) (Lee et al. 2008).

In addition, an autoregulation mechanism was proposed for Tgr in which maltotriose could be identified as a potential physiological effector (Kanai et al. 2007). The mechanism of autoregulation was already characterized for Lrs14 from *Sulfolobus solfataricus* (Bell and Jackson 2000) and an Lrp-like transcriptional regulator from *P.* *furiosus* (Brinkman et al. 2000). However, Tgr controls the currently largest regulon in Archaea, the Thermococcales glycolytic regulon with more than 30 genes (Kanai et al. 2007).

**Fig. 7:**
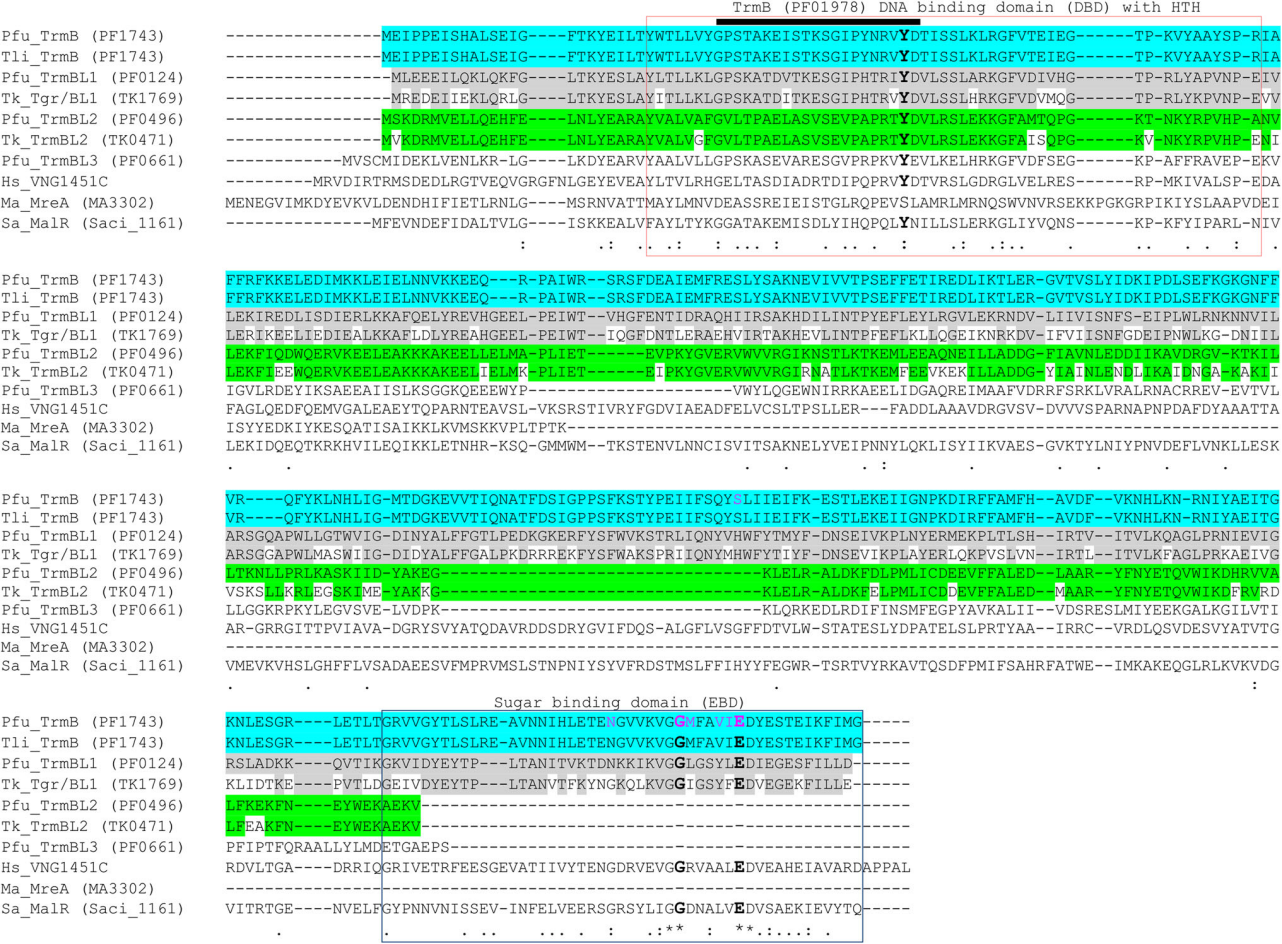
Sequence alignment of the discussed TrmB homologs. The *boxes* represent conserved protein domains. *Dashes* indicate gaps in the alignment. Highly conserved amino acids are represented via *asterisks*. *Dots* represent conserved amino acids. Strongly conserved amino acids with special function are highlighted in *bold letters*. The seven sugar binding amino acids of *Pfu* TrmB are shown in *pink letters*. Organism abbreviations are as follows: Pfu, *Pyrococcus* *furiosus*; Tli, *Thermococcus* *litoralis*; Tk, *Thermococcus* *kodakaraensis*; Hs, *Halobacterium* *salinarum*; Ma, *Methanosarcina* *acetivorans*; Sa, *Sulfolobus* *acidocaldarius*

Both, TrmBL1 and Tgr, consist of 341 amino acids and have a molecular weight of 39.4 kDa (Table 1). TrmBL1 appears as tetramer and octamer in a balanced state but addition of maltose or maltotriose as well as a high protein concentration shifts the equilibrium to the octameric form (Lee et al. 2007b). An alignment of their N-terminal amino acid sequences revealed a highly conserved α-helix with tyrosine at position 49 being essential for DNA recognition (Fig. 7) (Lee et al. 2007b). Moreover, an alternative sugar binding affinity and specificity is proposed for TrmBL1 and Tgr in comparison to TrmB because just two out of seven amino acids which are responsible for sugar binding in TrmB are conserved at the C-terminus (Gly_320_ and Glu_326_ in *P.* *furiosus*, Gly_324_ and Glu_330_ in *T.* *kodakarensis*). However, the amino acids flanking the sugar contacting ones of TrmB are conserved in both indicating a similar sugar binding pocket in all three proteins (Lee et al. 2007b).

**Fig. 8:**
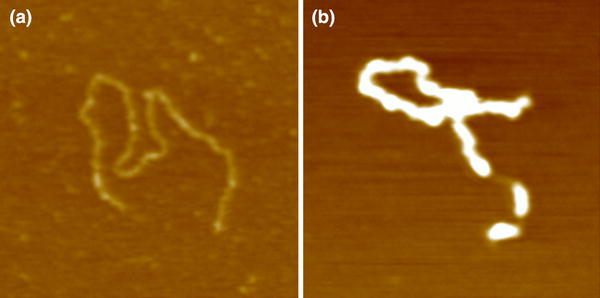
AFM images of a 3-kbp linear DNA of *Escherichia coli* (plasmid Bluescript II linearized by HindIII digestion). Image (**a**) shows the DNA without protein. Image (**b**) illustrates the DNA incubated with recombinant TK0471/TrmBL2 at a protein-to-DNA ratio of 10:1 (wt/wt) (taken from Maruyama et al. 2013 with permission from the authors and the publisher)

TrmBL2 is the most conserved protein of the TrmB family among the Thermococcales (Table 2). TK0471 of *T.* *kodakarensis* shows an 82 % sequence identity to *Pfu* TrmBL2. Furthermore, it also consists of 264 amino acids, but has a molecular weight of 30.8 kDa in contrast to *Pfu* TrmBL2 with 30.6 kDa (Table 1). A closer look at the highly conserved N-terminal DBD of both proteins reveals a HTH motif with tyrosine at position 50 being important for DNA recognition. Moreover, a C-terminal EBD is missing in both proteins (Fig. 7). Unlike Tgr, the *Pfu* TrmBL2 homolog TK0471 is binding to both coding and intergenic regions thereby repressing transcription when bound to promoter regions (Maruyama et al. 2011). Furthermore, it seems to play a major role as general chromosomal protein that is involved in agglutinating DNA to thick fibrous structures (Fig. 8) (Maruyama et al. 2011). Recent EMSA and ChIP-Seq experiments in our laboratory revealed that TrmBL2 seems to have a similar DNA-binding behavior as TK0471 of *T.* *kodakarensis* (data not shown). In contrast, nothing is known about the function of TrmBL2 in *P.* *horikoshii* (PH0799) and *P.* *abyssi* (PAB0838) so far, but the high identity of their amino acid sequences (92 and 91 %) to *Pfu* TrmBL2 indicates a similar function.

Furthermore, there are two additional members of the Thermococcales TrmB family with unknown function, TrmBL3 (PF0661) in *P.* *furiosus* and TrmBL4 (PH0751) in *P.* *horikoshii*. Sequence alignments argue for individual proteins within these organisms. In the case of TrmBL3 the N-terminal DBD is present whereas the C-terminal EBD is missing (Fig. 7) (Lee et al. 2007b).

### TrmB in *Halobacterium salinarum*

The haloarchaeal TrmB ortholog VNG1451C controls approximately 113 promoters in the absence of glucose or glycerol to directly regulate genes in diverse processes like the central carbon and amino acid metabolism with biosynthesis of the cognate cofactors, vitamin and purine biosynthesis as well as some more enzyme-coding genes in response to changes in carbon source availability. These genes are either unique in Archaea or conserved across all domains of life (Schmid et al. 2009). In addition, VNG1451C is a bifunctional regulator which is involved in the regulation of the redox and energy status of the cell in response to nutrient availability. Surprisingly, no binding was observed in the presence of high glucose or glycerol concentrations. Furthermore, VNG1451C bound to intergenic regions upstream of five transcription factors, including its own promoter (Table 1), what is contributing to a differential regulation of genes which are not directly influenced by the protein itself (Schmid et al. 2009).

VNG1451C consists of 360 amino acids with a calculated molecular mass of 39.4 kDa. Its N-terminus contains a winged HTH motif with tyrosine at position 61 possibly being essential for DNA binding like in *Pfu* TrmB (Fig. 7). Furthermore, VNG1451C shares a 21 % identity to the consensus sequence of the TrmB family signature (Pfam-ID: PF01978). ClustalW analysis showed that VNG1451C has three active site residues (Gly_337_, Glu_343_ and Asp_344_) which are critical for sugar binding in the characterized TrmB orthologs (Fig. 7) (Krug et al. 2006; Kanai et al. 2007; Lee et al. 2008; Schmid et al. 2009) and therefore also seems to code for a putative sugar binding transcriptional regulator. Interestingly, the gene locus coding for VNG1451C does not contain genes for the maltose and/or trehalose ABC transporters like in thermophilic Archaea. Nonetheless, VNG1451C seems to be a highly conserved regulator with a putative function related to sugar metabolism and the preservation of redox balance (Schmid et al. 2009). Phenotypic analysis of a VNG1451C knockout mutant in absence of glucose revealed a vehement growth defect in the mutant, even in rich media. In addition, the NAD^+^/NADH ratio was lower than in the wildtype strain. However, the growth defect as well as the NAD^+^/NADH ratio imbalance was reversed by the addition of glucose to the growth media whereas glycerol just partially had a complementational effect. Hence, this regulator seems to have a nutrient specificity because it reacts on glucose and glycerol but not on sugars like galactose, maltose, raffinose or sucrose as well as on pyruvate (Schmid et al. 2009).

The binding motif of VNG1451C is a *cis*-regulatory element with the sequence TACT-N_7-8_-GAGTA (Schmid et al. 2009) and is completely different from other characterized TrmB-binding sites like the TGM (van de Werken et al. 2006). VNG1451C can act as both an activator, with its binding motif upstream of the promoter, and as a repressor with the binding motif downstream of the promoter (Fig. 3a–d). This model was already proposed for *Tk* Tgr and *Pfu* TrmBL1 (Kanai et al. 2007; Lee et al. 2008). Recent data from Todor et al. (2013) revealed that the cobalamin biosynthesis pathway seems to be regulated by both VNG1451C and its dependent secondary regulators. In contrast, the purine biosynthesis pathway seems to be co-regulated by a non-VNG1451C-dependent transcription factor. Taken together, the TrmB ortholog VNG1451C in *H.* *salinarum* plays an important role in recognizing the nutrient availability and hence seems to directly regulate central metabolic enzyme-coding genes but also collaborates with other regulators to control peripheral metabolic pathways (Todor et al. 2013).

### TrmB in *Methanosarcina acetivorans*

The protein MreA (MA3302, *Methanosarcina* regulator of energy-converting metabolism) was identified by sequence analysis as a member of the TrmB family (Reichlen et al. 2012). It consists of 139 amino acids with a calculated molecular mass of 15.9 kDa and therefore is the smallest representative of the TrmB family so far (Table 1). The DBD is located within the first 111 N-terminal residues and a sequence alignment with Tgr of *T.* *kodakarensis* indicates a similarity of 68 % (Reichlen et al. 2012). However, the usually conserved tyrosine is replaced by a serine. Furthermore, a regulator domain at the C-terminus is missing like in *Pfu* TrmBL2 and TrmBL3 (Fig. 7) (Lee et al. 2007b). MreA is a global regulator of distinct methanogenic pathways (Reichlen et al. 2012). On the one hand, it represses genes which encode enzymes unique to pathways of methanogenesis from methylotrophic substrates; on the other hand, it activates genes encoding enzymes which are idiomatic in methanogenesis from acetate. Transcriptional profiling of wildtype versus *mreA* knockout strain revealed a diverse expression of 280 genes in acetate-grown cells. This indicates a key role of MreA in the regulation of specific genes essential for growth with acetate. A certain binding motif like the TGM of Thermococcales could not be identified.

### Members of the TrmB family in Crenarchaeota

Contrary to the Euryarchaeota, in the phylum Crenarchaeota only three organisms contain TrmB and TrmB-like transcriptional regulators: *Sulfolobus acidocaldarius, Caldivirga maquilingensis* and *Thermophilum pendens*. In *S.* *acidocaldarius* the best studied regulator is MalR (Saci_1161, mal regulon activator)—an activator of the maltose regulon (Wagner et al. 2013). According to Maruyama et al. (2011) also *C.* *maquilingensis* encodes two different TrmBs in its genome—Cmaq_1188 (related to MalR of *S.* *acidocaldarius*) and Cmaq_0601 (related to TrmBL4 of *P.* *horikoshii*) (http://www.archaea.ucsc.edu). In *T.* *pendens* four members of the TrmB family with both a DBD and an EBD domain similar to the TrmB domains were identified via phylogenetic analysis by Maruyama et al. 2011. However, the function of these proteins in *Caldivirga* and *Thermophilum* is not known.

### TrmBs in *Sulfolobus acidocaldarius*

MalR of *S.* *acidocaldarius* forms a separate group within the TrmB family because it is exclusively working as activator of the maltose regulon (Saci_1660-Saci_1666). It includes the ABC transporter *malEFGK*, an α-amylase *amyA* and an α-glucosidase *malA* (Choi et al. 2013). However, all other *Sulfolobus* species lack MalR homologs even though they retain a maltose transport regulon. This most likely indicates that *S.* *acidocaldarius* obtained the regulator by horizontal gene transfer. MalR consists of 349 amino acids and has a molecular weight of 40.3 kDa. Bioinformatic analysis of its amino acid sequence unveiled both a HTH domain with the conserved tyrosine at position 51 important for DNA binding and a TrmB EBD which comprises two amino acids essential for sugar binding in *Pfu* TrmBL1 (Gly_320_ and Glu_326_) (Fig. 7) (Lee et al. 2007b). The amino acid sequence identity with *P.* *furiosus* TrmB and TrmBL1 is 24.1 and 22.7 %, respectively. However, the expression of the genes of the maltose regulon was just induced by Mal R with maltose in the growth medium, indicating that MalR, in contrast to its TrmB homologues, is an activator of the *mal* gene cluster (Wagner et al. 2013).In addition, a MalR binding motif was detected upstream of the *malE* promoter with two 8-bp repeats (ATAATACT) located at −139 to −132 and at −106 to −99, but binding of MalR to these repeats was independent of the addition of sugars like maltose, D-glucose or D-xylose in quantitative RT-PCR and β-galactosidase activity assays. Furthermore, a feedback loop in which MalR regulates its own expression was proposed but unknown posttranscriptional processes or other regulators might also be involved in the regulation of MalR activity. All in all, MalR seems to be an individual regulator of maltose and maltodextrin transport components and/or metabolism, while sucrose transport and metabolism are not affected (Wagner et al. 2013).

### Members of the TrmB family in Thaum-, Nano- and Korarchaeota

Members of the TrmB family were also discovered in the remaining Archaeal phyla Thaum-, Nano- and Korarchaeota. Using phylogenetic sequence analysis one copy of TrmB could be detected in *Nitrosopumilus* *maritimus*, *Nanoarchaeum* *equitans* and Candidatus *Korarchaeum* *cryptofilum* (Maruyama et al. 2011). Until now, none of these TrmBs is specified.

## Conclusions

TrmBs of the different phyla in the Archaeal domain seem to play an important role in regulation of transcription of diverse metabolisms. Best studied are the TrmB proteins of the Thermococcales *P.* *furiosus, T.* *litoralis* and *T.* *kodakarensis*. The TrmBs of *H.* *salinarum, M.* *acetivorans* and of the Crenarchaeon *S.* *acidocaldarius* were also studied in some detail. A closer look at the protein structure of these TrmB proteins reveals that all proteins are able to bind to DNA using a HTH motif as DBD. The DBD is located at the N-terminal region and a mutational analysis revealed that is essential for binding. Most of the proteins contain in addition an EBD at the C-terminus with two conserved amino acids (Gly and Glu). Mostly, TrmBs also interact with inducers such as different sugars or other co-repressors or co-factors. Furthermore, all TrmBs seem to control diverse sugar transporters or different genes of sugar metabolism as well as genes involved in other metabolisms. Even the control of specific transcription factors via TrmB could be shown. In addition, they recognize different DNA-binding motifs which can be found upstream of the BRE/TATA box at promoters activated by their cognate TrmBs or downstream of the BRE/TATA box at promoters repressed by their TrmB representative. Whereas the euryarchaeal TrmBs seem to be highly conserved and frequently swapped via horizontal gene transfer, the crenarchaeal ones appear to have evolved in a different way.
